# A novel paradigm for cell and molecule interaction ontology: from the CMM model to IMGT-ONTOLOGY

**DOI:** 10.1186/1745-7580-6-1

**Published:** 2010-02-18

**Authors:** Francesco Pappalardo, Marie-Paule Lefranc, Pier-Luigi Lollini, Santo Motta

**Affiliations:** 1 Institute for Computing Applications 'M. Picone', National Research Council (CNR), Rome, Italy; 2 Faculty of Pharmacy, University of Catania, Catania, Italy; 3 IMGT, Laboratoire d'ImmunoGénétique Moléculaire, Institut de Génétique Humaine, UPR CNRS 1142, Montpellier, France; 4 Dipartimento di Ematologia e Scienze Oncologiche, Università di Bologna, Bologna, Italy

## Abstract

**Background:**

Biology is moving fast toward the virtuous circle of other disciplines: from data to quantitative modeling and back to data. Models are usually developed by mathematicians, physicists, and computer scientists to translate qualitative or semi-quantitative biological knowledge into a quantitative approach. To eliminate semantic confusion between biology and other disciplines, it is necessary to have a list of the most important and frequently used concepts coherently defined.

**Results:**

We propose a novel paradigm for generating new concepts for an ontology, starting from model rather than developing a database. We apply that approach to generate concepts for cell and molecule interaction starting from an agent based model. This effort provides a solid infrastructure that is useful to overcome the semantic ambiguities that arise between biologists and mathematicians, physicists, and computer scientists, when they interact in a multidisciplinary field.

**Conclusions:**

This effort represents the first attempt at linking molecule ontology with cell ontology, in IMGT-ONTOLOGY, the well established ontology in immunogenetics and immunoinformatics, and a paradigm for life science biology. With the increasing use of models in biology and medicine, the need to link different levels, from molecules to cells to tissues and organs, is increasingly important.

## Introduction

Biology is a knowledge-based discipline. Many predictions and interpretations of biological data are made by comparing the data against existing knowledge. Traditionally, the knowledge base in biology has resided within the heads of experienced scientists who have devoted much study and became experts in their particular domain. This approach worked well in the past, when considerable effort was needed to tease a few new data out of biological experiments. However, this situation is changing rapidly, and biology is moving fast toward the virtuous circle of other disciplines: from data to quantitative modeling and back to data. Models are usually developed by mathematicians, physicists, and computer scientists to translate qualitative or semi-quantitative biological knowledge into a quantitative approach [1].

To eliminate semantic confusion between biology and other disciplines, it is necessary to have a list of the most important and frequently used concepts coherently defined so that involved people could use such a set of definitions to create new models and software, to provide an exact, semantic specification of the concepts used in an existing schema and to curate and annotate existing database entries consistently. We notice here that it is important to understand that semantic ambiguities also can arise between human experts. However, in the course of a conversation usually enough background knowledge and context is available so that semantic ambiguities are most often faster resolved than even consciously recognized. This is possible because of our intelligent capabilities which computers, programs and databases, at least for the near future, fall yet short of.

An ontology describes basic concepts in a domain and defines relations among them. Basic building blocks of ontology design include concepts and their instances; properties of each concept describing various features and attributes of the concept (slots, sometimes called roles or properties); restrictions on slots (facets, sometimes called role restrictions). An ontology provides a common vocabulary for researchers who need to share information in the domain and allows to build knowledge databases. Ontologies are widely used in biology and medicine and several important ontology systems have been established. They contribute to a precise and exhaustive way to access bio-information and define concepts in a precise and rigorous way [2-9]. Interestingly, despite or because of the complexity of the immune response, IMGT-ONTOLOGY, the first ontology for immunogenetics and immunoinformatics, is also conceptually one of the more advanced biological ontologies [2-6], on which has been built IMGT^®^, the international ImMunoGeneTics information system^® ^http://www.imgt.org[10].

Other important efforts are underway to link models at different scales by means of markup languages (i.e. XML). CellML project is one of this http://www.cellml.org. The CellML language is an open standard based on the XML markup language. CellML is being developed by the Auckland Bioengineering Institute at the University of Auckland and affiliated research groups. The purpose of CellML is to store and exchange computer-based mathematical models. CellML allows scientists to share models even if they are using different modeling tools. It also enables them to reuse components from one model in another, thus accelerating model development.

Whereas usually ontologies led to knowledge databases, in what follows, we adopted another approach in which concepts for an ontology of cell and molecule interaction were generated starting from an agent based model (ABM), the Catania Mouse Model (CMM for short) and its computer implementation, the SimTriplex simulator [11,12]. SimTriplex simulates the immune system response elicited by the Triplex vaccine [13,14] against mammary carcinoma. This effort provides a solid infrastructure that is useful to overcome the semantic ambiguities that arise between biologists and mathematicians, physicists, and computer scientists, when they interact in such a multidisciplinary field.

**Figure 1:**
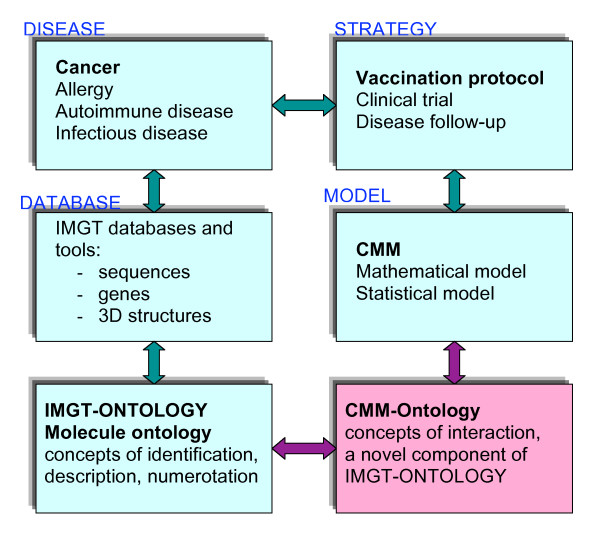
**Relations between the Molecule ontology of IMGT-ONTOLOGY that comprises concepts of identification, description and numerotation **[3-6]**and the CMM-Ontology that allowed to define concepts of interactions, a novel component of IMGT-ONTOLOGY (this paper)**. Whereas the concepts of identification, description and numerotation were defined to manage data in IMGT databases and tools and therefore immunogenetic knowledge in normal and pathological conditions (cancer, allergy, autoimmune disease...) [6,10], the concepts of identification were defined from mathematical and statistical models related to vaccination protocol [11,12].

The development of ontologies for molecular and cellular biology information, and the sharing of those ontologies within the bioinformatics community, are central problems in bioinformatics. If the bioinformatics community is to share ontologies effectively, ontologies must be exchanged in a form that uses standardized syntax and semantics. For this reason, while the initial motivation of our study was to present an ontology for the CMM, the paradigm we show here has wider applications as it bridges the molecule ontology with cell ontology (Figure 1). This is achieved by defining, at the same time, interactions in terms of cellular and molecular components of a biological system.

## Implementation

The Catania Mouse Model (CMM) has been developed using Unified Modeling Language (UML) http://www.uml.org/. UML is a diagramming language or notation to specify, visualize and document different types of models and object oriented software systems. UML helps in visualizing design and in communication. We used Umbrello UML Modeller http://uml.sourceforge.net, an open source tool that allows to manage and create UML based models. UML was selected because it is a widely-used system for the representation of objects and their relationships. Moreover the Umbrello tool was used to export the CMM classes into Extensible Markup Language/Resource Description Format (XML/RDF), in order to create the concepts of CMM-Ontology. XML was developed by the W3C http://www.w3.org. The current standard for the XML Schema Language is controlled by the XML Schema Working Group of the W3C. XML is a good candidate to share ontologies because of the significance of the Web and Web-based applications [15-17]. It is clear that the Web is rapidly becoming the primary method for the exchange of information and data, and that XML is currently the leading candidate for a generic language for the exchange of semi-structured objects.

### Distribution

CMM-Ontology main concepts, with controlled vocabularies and rules, are publicly available from the Computational Immunology and Immunomics Group homepage at http://www.immunomics.eu. Available formats are XML and XMI. The concepts of Identification for cellular components and the concepts of interaction have been added in IMGT-ONTOLOGY http://www.imgt.org.

#### From the model to the CMM-Ontology main concepts

CMM-Ontology concepts were generated from the model CMM. They provide a semantic standardization of the knowledge in the biological modeling field. They are used to identify the main biological entities used in the model as well as their interactions. We focus on two main types of concept: the concepts of identification and the concepts of interaction. These concepts bridge the gap between molecular component ontology and cellular component ontology. It is expected that they will allow scientists to easily identify the main biological entities they use, to model any given biological scenario.

### Concepts of Identification

**Figure 2:**
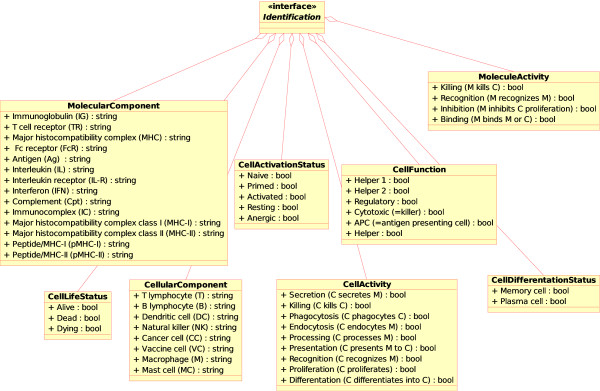
**Concepts of identification in CMM-Ontology**. These concepts have been added to IMGT-ONTOLOGY [6] for allowing identification of the component (MolecularComponent, CellularComponent), component status (CellLifeStatus, CellDifferentiationStatus, CellActivationStatus), activity (MoleculeActivity, CellActivity) and function (CellFunction). The name of the concepts of identification are shown in bold and the instances are shown with a prefixed '+" sign.

Concepts of identification for molecular components have been analyzed extensively in IMGT-ONTOLOGY [3-6]. We therefore focus, in CMM and CMM-Ontology, on the identification of the cellular entities involved in modeling the competition between cancer and the immune system with or without exogenous stimulation with a cancer vaccine. In CMM, and in accordance with UML, the classes have been developed as a "class diagram" (these classes correspond to concepts in CMM-Ontology). A class defines the attributes and the methods of a set of objects. All objects of a given class (instances of this class) share the same behavior, and have the same set of attributes (each object has its own set). In UML, classes are represented by rectangles, with the name of the class, and can also show the attributes and operations of the class in two other "compartments" inside the rectangle. Interfaces are abstract classes, which means that instances cannot be directly created inside them. They can contain operations but not attributes. An association represents a relationship between classes, and gives the common semantics and structure for many types of "connections" between objects. Associations are the mechanism that allows objects to communicate with each other. In UML, associations are represented as lines connecting the classes participating in the relationship. Aggregations are a special type of association in which the two participating classes do not have an equal status, but make a "whole-part" relationship. An aggregation describes how the class that takes the role of the whole, is composed of (has) other classes, which takes the role of the parts. In UML, aggregations are represented by an association line that ends in a diamond on the side of the whole. A generalization association between two classes puts them in a hierarchy representing the concept of inheritance of a derived class from a base class. In UML, generalizations are represented by a line connecting the two classes, with an ending arrow on the side of the base class. Containment associations represent an operation implementation. In UML, containments are represented by a line with a circle. In CMM-Ontology, the concepts of identification and their instances, generated from the model, are the following (Figure 2):

• MolecularComponent. This concept identifies the molecules. Instances of this concept are: immunoglobulin (IG), T cell receptor (TR), interleukin (IL), antigen (Ag), immunocomplex (IC), major histocompatibility complex (MHC), major histocompatibility complex class I (MHC-I), major histocompatibility complex class II (MHC-II), peptide/MHC-I (pMHC-I), peptide/MHC-II (pMHC-II), Fc receptor (FcR), interleukin receptor (IL-R), interferon (IFN), complement (Cpt).

• MoleculeActivity. This concept identifies activities mediated by molecules. Instances of this concept are binding, recognition, inhibition of proliferation, killing.

• CellularComponent. This concept identifies the cells. Instances of this concept are B lymphocyte (B), T lymphocyte (T), macrophage (M), natural killer (NK), dendritic cell (DC), cancer cell (CC), vaccine cell (VC), mast cell (MC).

• CellActivity. This concept identifies the activities that a cell can carry out. These activities can involve another cell and/or a molecule. Instances of this concept are detailed in Figure 2.

• CellActivationStatus. This concept identifies the activation status of a cell. Instances of this concept are: naive, primed, activated, resting, anergic.

• CellFunction. This concept identifies the functions that a cell can perform. Instances of this concept are: helper (helper 1, helper 2), regulatory, cytotoxic, antigen presenting cell (APC).

• CellDifferentiationStatus. This concept identifies the differentiation status of a cell. Instances of this concept are, for example, memory cell, plasma cell.

• CellLifeStatus. This concept identifies the life status of a cell and was specifically included for modeling purposes. Instances of this concept are: alive (i.e. a cell is performing its own job), dead (a cell to be removed from the system) or dying (a cell starting an apoptosis process and supposed to do some other actions before it dies, i.e. releasing of antigens or some other cell product).

### Concepts of interaction

An interaction between two entities is a complex action which eventually ends in a status change of one or both entities. In the immune system, interactions can be specific (adaptive immunity) or non specific (innate immunity). Specific interactions characterize the immune adaptive response and comprise a specific recognition phase between two entities, the antigen receptor and an antigen. These interactions involve the recognition of an antigen by:

• either an immunoglobulin (IG) specific for that antigen (in CMM, native p185 antigen). The antigen can be either soluble or adsorbed at the surface of a follicular dendritic cell in the lymph node;

• or a T cell receptor (TR) specific for a peptide/MHC. The peptide (p) resulting from antigen processing by a cell is presented at the surface of that cell in the groove of a MHC protein of class I or II (MHC-I or MHC-II) [18]. A TR is specific of a pMHC-I or pMHC-II (in CMM, peptides processed from p185 and presented by MHC-I or MHC-II). Immune recognition can be eventually enhanced by adjuvants.

In CMM, the interactions have been modeled using component diagrams. They show the components (either component technologies or sections of the system which are clearly distinguishable) and the artifacts they are made of, such as source code files, or relational database tables. Components can have interfaces (i.e. abstract classes with operations) that allow associations between components.

**Figure 3:**
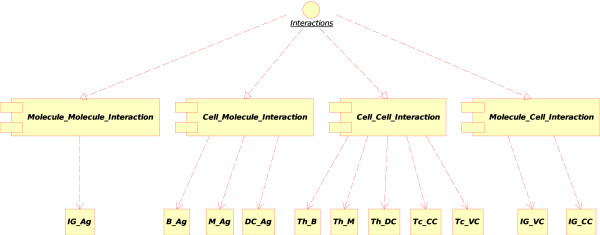
**Concepts of interactions in CMM-Ontology**. These concepts have been added to IMGT-ONTOLOGY [6] for defining interactions between molecules (Molecule_Molecule_Interaction), cells (Cell_Cell_Interaction) and between molecules and cells (Molecule_Cell_Interaction, Cell_Molecule_Interaction). Main instances of these concepts used in the CMM model are indicated: Immunoglobulin_Antigen (IG_Ag), B lymphocyte_Antigen (B_Ag), Macrophage_Antigen (M_Ag), Dendritic cell_Antigen (DC_Ag), T lymphocyte [helper]_B lymphocyte (Th_B), T lymphocyte [helper]_Macrophage (Th_M), T lymphocyte [helper]_Dendritic cell (Th_DC), T lymphocyte [cytotoxic]_Cancer cell (Tc_CC), T lymphocyte [cytotoxic]_Vaccine cell (Tc_VC), Immunoglobulin_Vaccine cell (IG_VC) and Immunoglobulin_Cancer cell (IG_CC).

**Figure 4:**
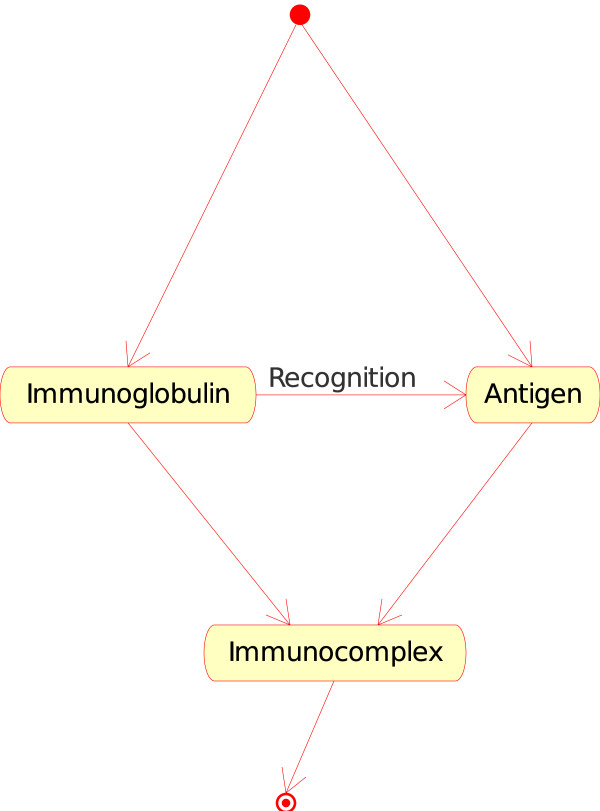
**Immunoglobulin_Antigen as an instance of the "Molecule_Molecule_Interaction" concept**. This instance is shown as IG_Ag in Fig. 2. In CMM, Ag is represented by p185.

**Figure 5:**
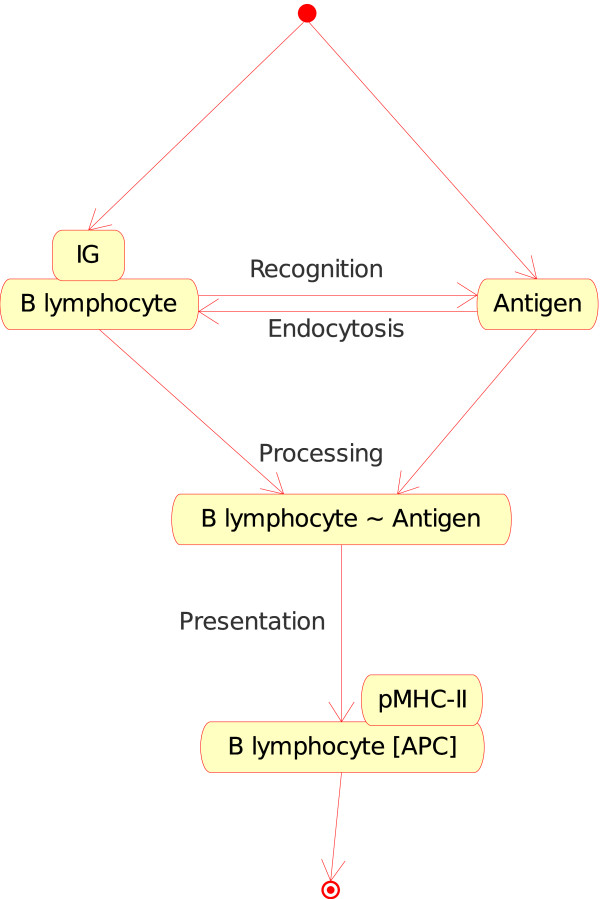
**B lymphocyte_Antigen as an instance of the "Cell_Molecule_Interaction" concept**. This instance is shown as B_Ag in Fig. 2. In CMM, Ag is represented by p185. Note that the interaction between the membrane IG, on the surface of the B lymphocyte, and the antigen is also an instance of the "Molecule_Molecule_Interaction". This example illustrates (as the following figures) how the IMGT-ONTOLOGY concepts of interactions allow to bridge the gap between the cell and molecule levels.

**Figure 6:**
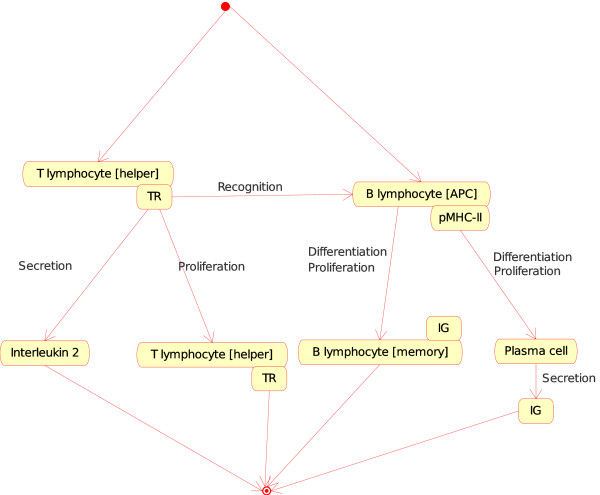
**T lymphocyte [helper]_B lymphocyte [APC] as an instance of the "Cell_Cell_Interaction" concept**. This instance is shown as Th_B in Fig. 2. Note that the interaction between the T cell receptor (TR), on the surface of the T lymphocyte [helper], and the pMHC-II, on the surface of the B lymphocyte (as antigen presenting cell or [APC]), is also an instance of the "Molecule_Molecule_Interaction". This example illustrates, as Fig. 4, how the IMGT-ONTOLOGY concepts of interactions allow to bridge the gap between the cell and molecule levels.

In CMM-Ontology, the concepts of interaction and their instances, generated from the model, are the following (Figure 3):

1. The "Molecule_Molecule_Interaction" concept. If the Molecule is a soluble immunoglobulin (IG) specific for an antigen, and if the other Molecule encountered is that antigen (Ag), IG binds to Ag and forms an immunocomplex (that can be captured by a macrophage). That instance of the "Molecule_Molecule_Interaction" concept is

• Immunoglobulin_Antigen. In CMM, Ag is the native p185 antigen (Figure 4).

2. The "Cell_Molecule_Interaction" concept. If the Cell is a B lymphocyte, a macrophage or a dendritic cell, and if the Molecule is an antigen, the cell can internalize the native antigen, process it and present it as peptide bound to MHC-II (pMHC-II) protein at the cell surface. The cell becomes a professional antigen presenting cell (or APC). Three instances can therefore be defined:

• B lymphocyte_Antigen (Figure 5). If, in a lymph node, a naive B lymphocyte expresses at the cell surface a membrane IG which is specific for the native antigen (in CMM, p185 antigen), B lymphocyte internalizes the membrane IG and the bound Ag and processes the IG-Ag complex into peptides which are then presented by MHC-II (pMHC-II) at the B lymphocyte surface. B lymphocyte becomes an APC.

• Macrophage_Antigen. If a macrophage encounters a native antigen (in CMM-Ontology, p185 antigen) or an immunocomplex, the macrophage internalizes the antigen or the immunocomplex and processes it into peptides which are then presented by MHC-II (pMHC-II) at the macrophage cell surface. Macrophage becomes an APC.

• Dendritic cell_Antigen. If a naive dendritic cell encounters a native antigen (in CMM, p185 antigen) or an immunocomplex, the dendritic cell internalizes the antigen or the immunocomplex and processes it into peptides which are then presented by MHC-II (pMHC-II) at the dendritic cell surface. Dendritic cell becomes an APC.

3. The "Cell_Cell_Interaction" concept.

(a) If, in a lymph node, one Cell is a T lymphocyte [helper] (Th) and the other Cell is a B lymphocyte [APC], the T cell (identified as CD4+) becomes an activated T helper lymphocyte that helps the B cell to differentiate into plasma cell or memory cell (Figure 6) At the molecular level, the T cell receptor (TR) at the surface of the T lymphocyte [helper] (Th) binds specifically pMHC-II at the surface of the B lymphocyte [APC], Th proliferates and secretes interleukin 2 (IL2). At the same time, B lymphocyte proliferates and differentiates into a plasma cell (that secretes IG) or into a memory cell (with IG at its surface). That instance of the "Cell_Cell_Interaction" concept is:

• T lymphocyte [helper]_B lymphocyte [APC] (Figure 6). Two other instances involving a Th and an APC are the following:

-T lymphocyte [helper]_Macrophage [APC];

-T lymphocyte [helper]_Dendritic cell [APC]. Following interaction with Macrophage [APC] or Dendritic cell [APC], Th becomes activated and secretes interleukins that activate other cells of the immune response (NK, mast cells, T cytotoxic lymphocytes...).

**Figure 7:**
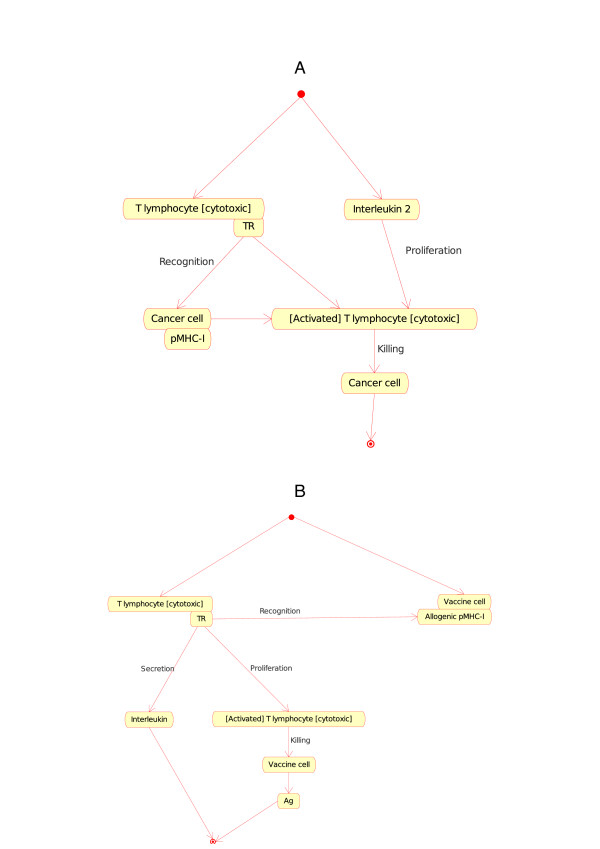
**T lymphocyte [cytotoxic]_Cancer cell (7A) and T lymphocyte [cytotoxic]_Vaccine cell (7B) as instances of the "Cell_Cell_Interaction" concept in models related to immunotherapy, as defined in CMM**. These instance are shown as Tc CC and Tc VC in Fig. 2. The interaction between the T cell receptor (TR), on the surface of the T lymphocyte [cytotoxic], and the pMHC-I, on the surface of the cancer cell or vaccine cell, is also an instance of the "Molecule_Molecule_Interaction". It represents an additional example of interactions that bridges the gap between cell and molecule levels in the adaptive immune response (IMGT Education, http://www.imgt.org) [10].

**Figure 8:**
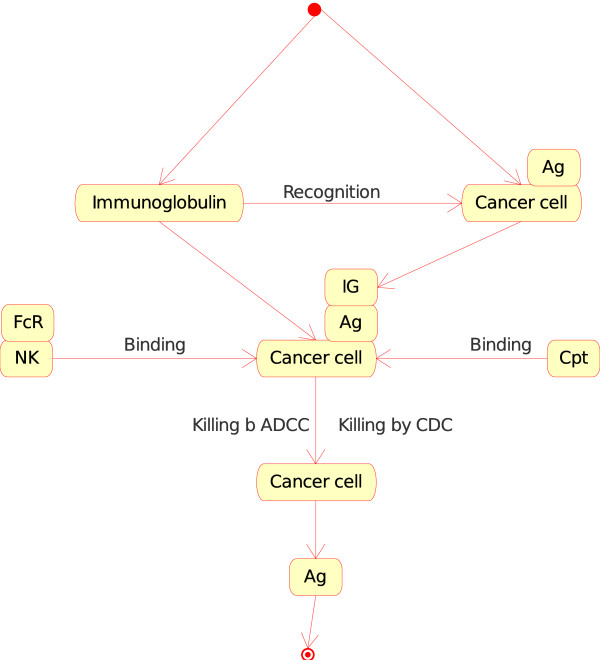
**Immunoglobulin_Cancer cell as an instance of the "Molecule_Cell_Interaction" concept in models related to immunotherapy, as defined in CMM**. This instance is shown as IG_CC in Fig. 2. The interaction between the immunoglobulin and the antigen (Ag), on the surface of the cancer cell (in CMM, Ag is represented by p185), is the starting event that leads to effector interactions: (i) binding, at the cell level, of natural killer (NK) to cancer cell ("Cell_Cell_Interaction") and, at the molecule level, of the Fc receptor (FcR), on the surface of NK, to the IG that has recognized Ag on the cancer cell surface ("Molecule_Molecule_Interaction") and (ii) binding of the complement (Cpt) to the IG that has recognized Ag on the cancer cell surface ("Molecule_Molecule_Interaction"). These interactions by NK or complement lead to the killing of the cancer cell by 'antibody dependent cell cytotoxicity' or ADCC or 'complement dependent cytotoxicity' (CDC), respectively (IMGT Education > IMGT Lexique, ADCC and CDC, http://www.imgt.org).

(b) If one Cell is a T lymphocyte [cytotoxic] (Tc) and the other Cell is a cancer cell (or a vaccine cell, a characteristic cell of the CMM-Ontology), the T cell (identified as CD8+) becomes, in presence of IL2, an activated T cytotoxic lymphocyte that kills the other cell (cancer cell or vaccine cell). At the molecular level, the T cell receptor (TR) at the surface of a naive T lymphocyte [cytotoxic] (Tc) binds specifically pMHC-I at the surface of the cell (cancer cell or vaccine cell), in the presence of IL2, T lymphocyte [cytotoxic] (Tc) is activated and kills the other cell (cancer cell or vaccine cell). The two corresponding instances of the Cell_Cell_Interaction concept are:

• T lymphocyte [cytotoxic]_Cancer cell (Figure 7A).

• T lymphocyte [cytotoxic]_Vaccine cell (Figure 7B).

4. The "Molecule_Cell_Interaction" concept. If the Molecule is a specific soluble immunoglobulin (IG) and the Cell is a cancer cell (or a vaccine cell) that expresses the antigen at its cell surface (in CMM, p185), the soluble IG recognizes specifically the antigen (Figure 8). The opsonized cell (cell with bound IG on its surface) may be killed by complement dependent cytotoxicity (CDC) or by antibody dependent cell cytotoxicity (ADCC). At the molecular level, the first interaction is the recognition by the IG of the antigen expressed at the surface of the cancer cell or vaccine cell. The two corresponding instances of the "Molecule_Cell_Interaction" concept are:

• Immunoglobulin_Cancer cell.

• Immunoglobulin_Vaccine cell.

The consequence of the "Molecule_Cell_Interaction" in these two instances, that results in the killing (by CDC or ADCC) of the opsonized cancer cell or vaccine cell involves new instances of concepts defined above:

• "Molecule_Cell_Interaction", for CDC (with the instance Complement_Opsonized cell), or

• "Cell_Cell_Interaction", for ADCC (with the instance Natural killer_Opsonized cell).

In CDC, complement (C1q) binds to the Fc of IG at the surface of the opsonized cell (cancer cell or vaccine cell), the complement cascade is activated, the membrane attack complex (MAC) is formed in the cell membrane and the cell is killed. In ADCC, Fc receptor gamma of natural killer cell (NK) binds to the Fc of IG at the surface of the opsonized cell (cancer cell or vaccine cell), NK kills the cell (cancer cell or vaccine cell).

## Discussion

An interaction described at the "Cell_Cell_Interaction" level focuses on the activity, activation status, function, differentiation status and/or life status of the cells that interact. If needed, the "Cell_Cell_Interaction" can also be identified at the "Molecule_Cell_Interaction", or "Cell_Molecule_Interaction", or "Molecule_Molecule_Interaction" levels. The level of granularity will depend on the model and on the kind of data that are available for the modeling. The four types of interactions can be used to identify, not only a given interaction, but also a complex succession of interactions, as described above for the killing by CDC or ADCC. Finally we have shown that these concepts of interactions identified in CMM-Ontology are general for the immune response and, for that reason, they have been added to IMGT-ONTOLOGY [6], the reference in immunogenetics and immunoinformatics. This effort represents, as far we know, the first attempt at linking the well established molecular ontology with cellular ontology in cancer immunology. With the increasing use of models in biology and medicine the need of linking different levels, from molecules to cells to tissues and organs, is increasingly important. As a matter of fact only a unified ontology framework will allow to link models at different scales.

Attempts in that direction are, at the moment, based on the use of markup languages [15-17], i.e. XML, but a general framework is still to come. In modeling other pathologies [19-21] we experienced that using an ontology driven approach, itself generated from a model, resulted in speeding up the process of model construction as well as clarifying the biologist needs regarding model definion. We believe that this is a powerful methodology.

A model is a formal description of biological knowledge and its quantitative formulation using mathematical or computational tools. An ontology based description of these tools would clarify to biologists the value of the model results. However very few attempts in these directions have been made for modeling in physics and engineering [22]. We expect that the increasing interest in mathematical modeling in life science will push toward an increasing interest in this aspect in a near future.

## Conclusion

In this paper we presented a first attempt, generated from a model, at defining an integrated molecular -cellular ontology to be used in modeling biological problems. As the overall goal of this approach is to use a standardized approach to describe biological entities we plan to adapt in the future this methodology to the most widely used software tool in this field, i.e. Protégé http://protege.stanford.edu). Work in this direction is in progress and results will be published in due course.

## Competing interests

The authors declare that they have no competing interests.

## Authors' contributions

FP and SM took care of agent based model related concepts and relations ontologies and provided the link to molecular and cellular ontology. FP designed the UML model using Umbrello software. MPL took care of the ontology related issues, in particular she contributed to molecular and cellular concepts of the ontology and bridging them through their interactions. PLL contributed general immunological knowledge and specific know-how and experimental results of in vivo tumor immunology in HER-2/neu transgenic mice. All authors read and approved the final manuscript.

## References

[B1] Bodenreider O Stevens R Bio-ontologies: current trends and future directions Briefings in Bioinformatics 2006 7 3 256 274 10.1093/bib/bbl027 16899495 PMC1847325

[B2] Lefranc MP Giudicelli V Regnier L Duroux P IMGT, a system and an ontology that bridge biological and computational spheres in bioinformatics Briefings in Bioinformatics 2008 9 4 263 75 10.1093/bib/bbn014 18424816

[B3] Giudicelli V Lefranc MP Ontology for Immunogenetics: IMGT-ONTOLOGY Bioinformatics 1999 15 1047 1054 10.1093/bioinformatics/15.12.1047 10745995

[B4] Lefranc MP Giudicelli V Ginestoux C Bosc N Folch G Guiraudou D Jabado-Michaloud J Magris S Scaviner D Thouvenin V Combres K Girod D Jeanjean S Protat C Monod MY Duprat E Kaas Q Pommié C Chaume D Lefranc G IMGT-ONTOLOGY for Immunogenetics and Immunoinformatics In Silico Biology 2004 4 17 29 15089751

[B5] Lefranc MP Clément O Kaas Q Duprat E Chastellan P Coelho I Combres K Ginestoux C Giudicelli V Chaume D Lefranc G IMGT-Choreography for Immunogenetics and Immunoinformatics In Silico Biology 2005 5 45 60 15972004

[B6] Duroux P Kaas Q Brochet X Lane J Ginestoux C Lefranc MP Giudicelli V IMGT-Kaleidoscope, the Formal IMGT-ONTOLOGY paradigm Biochimie 2008 90 570 583 10.1016/j.biochi.2007.09.003 17949886

[B7] The Gene Ontology Consortium Gene ontology: tool for the unification of biology Nat Genet 2000 25 25 29 10.1038/75556 10802651 PMC3037419

[B8] Kanehisa M Araki M Goto S Hattori M Hirakawa M Itoh M Katayama T Kawashima S Okuda S Tokimatsu T Yamanishi Y KEGG for linking genomes to life and the environment Nucleic Acids Res 2008 36 D 480 484 10.1093/nar/gkm882 PMC2238879 18077471

[B9] Reyes-Palomares A Montañez R Real-Chicharro A Chniber O Kerzazi A Navas-Delgado I Medina MA Aldana-Montes JF Sánchez-Jiménez F Systems biology metabolic modeling assistant: an ontology-based tool for the integration of metabolic data in kinetic modeling Bioinformatics 2009 25 6 834 5 http://bioinformatics.oxfordjournals.org/cgi/content/full/25/6/834 10.1093/bioinformatics/btp061 19189977

[B10] Lefranc MP Giudicelli V Ginestoux C Jabado-Michaloud J Folch G Bellahcene F Wu Y Gemrot E Brochet X Lane J Regnier L Ehrenmann F Lefranc G Duroux P IMGT^®^, the international ImMunoGeneTics information system ^®^ Nucleic Acids Res 2009 37 D 1006 1012 10.1093/nar/gkn838 PMC2686541 18978023

[B11] Pappalardo F Castiglione F Lollini PL Motta S Modeling and simulation of cancer immunoprevention vaccine Bioinformatics 2005 21 12 2891 2897 10.1093/bioinformatics/bti426 15817697

[B12] Motta S Lollini PL Castiglione F Pappalardo F Modelling vaccination schedules for a cancer immunoprevention vaccine Immunome Research 2005 1 5 [Doi:10.1186/1745-7580-1-5] 16305756 10.1186/1745-7580-1-5 PMC1287065

[B13] Lollini PL De Giovanni C Pannellini T Cavallo F Forni G Nanni P Cancer immunoprevention Future Oncology 2005 1 57 66 10.1517/14796694.1.1.57 16555976

[B14] Lollini PL Cavallo F Nanni P Forni G Vaccines for tumour prevention Nature Reviews Cancer 2006 6 3 204 216 10.1038/nrc1815 16498443

[B15] Chaume D Giudicelli V Lefranc MP IMGT-ML a XML language for IMGT-ONTOLOGY and IMGT/LIGM-DB data. CORBA and XML : Towards a Bioinformatics Integrated Network Environment Proceedings of NETTAB 2001, Network Tools and Applications in Biology 2001 71 75

[B16] Chaume D Combres K Giudicelli V Lefranc MP Retrieving factual data and documents using IMGT-ML in the IMGT information system. Technologies and technological platforms of interest to the field, with emphasis on: Ontologies, Databases and Applications of Semantics in Bioinformatics NETTAB 2005 Work ows management: new abilities for the biological information overflow 2005 47 51

[B17] Hunter PJ Crampin EJ Nielsen PMF Bioinformatics, multiscale modeling and the IUPS Physiome Project Briefings in Bioinformatics 2008 9 4 333 343 10.1093/bib/bbn024 18477639

[B18] Lefranc MP Duprat E Kaas Q Tranne M Thiriot A Lefranc G IMGT unique numbering for MHC groove G-DOMAIN and MHC superfamily (MhcSF) G-LIKE-DOMAIN Dev Comp Immunol 2005 29 917 938 10.1016/j.dci.2005.03.003 15936075

[B19] Pappalardo F Musumeci S Motta S Modeling immune system control of atherogenesis Bioinformatics 2008 24 15 1715 1721 10.1093/bioinformatics/btn306 18556669

[B20] Pappalardo F Motta S Lollini PL Mastriani E Analysis of vaccine's schedules using models Cellular Immunology 2007 244 137 140 10.1016/j.cellimm.2007.03.002 17442286

[B21] Castiglione F Pappalardo F Bernaschi M Motta S Optimization of HAART by means of Genetic Algorithms and agent based models of HIV infection Bioinformatics 2007 23 24 3350 3355 10.1093/bioinformatics/btm408 17942443

[B22] Thomas R Olsen G Olsen GR Jon Doyle, Piero Torasso, Erik Sandewall An Ontology for Engineering Mathematics, Volume Fourth International Conference on Principles of Knowledge Representation and Reasoning 1994 Morgan Kaufman

